# A comparison of global surgery tariffs and the actual cost of bills at Hazrate Rasoole Akram educational and medical center

**DOI:** 10.1186/s12962-020-00232-w

**Published:** 2020-09-29

**Authors:** Ali Aboutorabi, Maryam Radinmanesh, Aziz rezapour, Mahnaz afshari, Ghasem taheri

**Affiliations:** 1 Department of Health Economics, School of Health Management and Information Sciences, Iran University of Medical Sciences, Tehran, Iran; 2 Health Management and Economics Research Center, Iran University of Medical Sciences, Tehran, Iran

**Keywords:** Global surgery, Health service tariff, Iran, Medical expenses

## Abstract

**Background:**

the health service tariff is an appropriate policymaking tool and the financial leverage of the health system control which affects quality, availability, cost, efficiency, equity and accountability of health services. Global surgeries include 91 common cases of general and specialized surgeries in hospitals; fixed tariffs are annually defined for these surgeries, and insurance companies must pay medical centers based on these tariffs. The aim of this study was to examine and compare hospital bills with global surgery tariffs at Hazrate Rasoole Akram Educational and Medical Center in 2017.

**Methods:**

This descriptive-analytic study was conducted retrospectively and compared the global and actual costs of global surgeries performed in the third quarter of the year 2017 at Hazrate Rasoole Akram Educational and Medical Center. Required data on the actual costs of surgeries was collected through the Hospital Information System (HIS) and patients’ records. Information on the global costs was obtained from the Annual Circulars of Insurance Council for the studied period about the cost of global surgeries. Linear regression (STATA13 software) was used to investigate the effect of items on tariff and invoice differences; concerning other calculations, EXCEL software was used.

**Results:**

The highest frequency of global surgeries was related to ophthalmic surgery which accounted for approximately half of total surgeries performed at Hazrate Rasoole Akram Hospital. The most significant difference between global tariff and invoice was also related to ophthalmic surgery (188709.3 Dollar a year).Overall, the actual hospital bills were much higher than the tariffs approved for global surgeries, and the total difference was 461805.5 Dollar. The results revealed that there was a significant relationship between some of the items such as the cost of operating rooms, anesthesia and other services.

**Conclusions:**

Referral hospitals which are at the level three of referral networks usually treat more complex patients; this should be taken into account when defining surgery tariffs of these centers. On the other hand, hospitals need to control the costs and reduce the end cost of these surgeries by improving clinical management and cost management. In addition, prospective and case-based payment methods can control health costs.

## Background

The considerable increase in health and treatment costs has made governments and organizations analyze these services from a financial and economic point of view [1–3].also Financial resources are an essential input to health systems [4] and in this way Huge hospital expenses and financial constraints in Iran have caused the government and health authorities to be more aware of low efficiency of hospital beds and to pay more attention to hospital management [5] and they know more money alone will not solve the problem [1]. Therefore, proposing practical plans to increase the efficiency and to reduce the cost of hospital beds has been put on Iran’s health system agenda [5].One of the most important ways to control health care costs in different insurance systems is to use an appropriate payment system for health care providers and suppliers [5]. Studies have revealed that each of the existing systems has its own strengths and weaknesses; some of them are important in terms of efficiency and some in terms of effectiveness. For example, one of the methods of paying medical expenses is a “free for service” system which increases physicians’ motivation. The result of this payment system is that service providers have sufficient incentives to irrationally increase the services and induced demand for the patients and thereby to increase medical costs [6]. A study by Gerdtham et al. showed that the “free for service” payment system led to an 11% increase in health costs in OECD countries [7]. Therefore, designing a plan according to which payment must be made for all services provided to.

A patient using the DRG system is considered a significant shift from the “free for service” payment system in all countries. The DRG system is a system used to pay a fixed amount for special patients based on a diagnostic group rather than the cost of each service [8].

None of the payment methods is complete alone and each has its own advantages and disadvantages. Therefore, synthetic methods are used in many countries. In Iran, a combination of three methods “budget payment, free for service and case payment (Global)” is used [9]. The relative value of hospital services in Iran is extracted from the book “the Relative Value” using the free for service method. From 1993 to 2014, the book “the Relative Values of the American Health Services, known as the California Book” was used to determine the relative value of health services in Iran. However, this book was considered unrealistic due to the outdated nature of the book and therapeutic methods as well as the addition of some of the modern therapeutic methods and technologies. In 2014 in line with the goals of the Ministry of Health, health service tariffs were revised and updated in the Health System Reform Plan in order to increase health service coverage, to reduce out-of-pocket payments and to increase the incentives of health service providers, especially medical experts [10].

Moreover, the Higher Insurance Council at the Ministry of Health, Treatment and Medical Education adopted a resolution on applying tariffs on aggregate calculation (global) on the forty-third session on 02/28/1999; subsequently, 60 common surgeries were announced (91 cases now). Article one of the executive regulation of the plan states that aggregate calculation tariffs (global) have been determined based on a review of 65,000 patients’ records (both hospitalized and discharged) and their costs, taking into account cases such as patient’s age and other potential services. Therefore, the total tariff is the actual average value of services provided to patients [11].

Every year, large amounts of capitals of insurance organizations are lost due to the prescription of unnecessary medical services by the physicians and medical centers, prolonged hospitalization and spending too much resources on reviewing hospital bills and documents. Since the “free for service” payment system has many shortcomings in practice, a research was carried out in 1997 to determine the general payment tariff for common surgical procedures. The basis for the payment of medical costs in this system (general or global tariff), which is in fact an extraction of the DRG payment system, is based on the treatment of each patient rather than providing a specific service. In other words, this payment system pays a certain amount for the treatment of patients, depending on the type of illness, in order to control costs and make good use of resources; this amount has been predetermined for the hospital. This amount which is based on the resources needed to treat each disease stops providers to increase unnecessary services [11].

Now, after several years of implementing this plan, studies have shown that global service tariffs are lower than the end cost for hospitals, resulting in hospital loss, increased unnecessary referral burdens and unnecessary payments [12–15]. The aim of this study was to compare the global surgery tariffs with the real costs in Hazrate Rasoole Akram Educational and Medical Center in 2017.

## Methods

This descriptive-analytic study was conducted retrospectively and compared the global costs and costs recorded on the bills of patients hospitalized for all global surgeries in the third quarter of the year 2017 at Hazrate Rasoole Akram Educational and Medical Center. The reason for this choice is that in the third quarter of the year, the global tariffs are fully implemented in hospitals for the new year.

The statistical population of this study includes all records of hospitalized patients undergoing global surgery in the period of October, November and December of 2017 at Hazrate Rasoole Akram Hospital. The sampling method was of census type. The data collection tool used in this study was a form designed by the researchers; it included some information such as admission type, admission and discharge date, the physician, the type of basic insurance, costs of the global file submitted to the insurance company and cost information related to the costs recorded on patients’ bills based on the Ministry of Health tariffs. Cost information included costs of the operating room, laboratories, beds, surgeons, surgeons’ assistance, nursing services, anesthesia, drugs, technical fees, consumables, radiology, operating room medicine, operating room consumables, consulting, other services, global tariff, patient franchise, subsidy and insurers’ share.

In the first phase, the required information on global tariffs and the title of global surgeries was obtained from the Annual Report of the Supreme Insurance Council in 2017. The list of global surgeries performed at Hazrate Rasoole Akram Hospital was then identified by referring to the Hospital Revenue Department. Subsequently, data was collected through the Hospital Information System (HIS) and patients’ records using a designed form and by referring to the Hospital Health Information Technology and Management Department. For all hospitalized patients, all bill information sent to insurance agencies as well as information about patients’ cost bills was collected in terms of cost items. Inpatients’ records were reviewed to ensure the accuracy of the information and to ensure that the information recorded in the information system was accurate and exact. The global tariff information was also reconciled with the Supreme Council Circular and its accuracy was assessed.

In the second phase, global surgeries were classified in terms of specialties:1- Ophthalmic surgeries 2- Gynecological surgeries- Neurosurgery 4- General surgeries 5- Orthopedic surgeries 6- Urology surgeries 7- ENT surgeries and 8- cardiac surgeries.

Cost information of total cost of medication including operating room and ward medications, total cost of consumables including operating room and ward consumables, total cost of surgery including surgeon premiums, surgeons’ assistance and consulting fees and total para-clinical cost was aggregated to avoid the probable coincidence in analyses and to analyze it in the software. An item called actual cost difference of hospital and global bills was also extracted from the difference between the total hospital bills and the global bills and was used as the dependent variable in the analyses.

The data was analyzed descriptively and analytically. In the descriptive section, the frequency, sum, maximum, minimum and average difference between global tariffs and hospital bills were examined by excel software in terms of surgeries. Moreover, the study variables (insurance type, surgery type specialty, length of hospital stay, tariff difference, bills and other items) were estimated using STATA version 22. In the analytical section, given the normality of the variables, linear regression model was used to determine the factors affecting the difference between the tariff and the bill. STATA software was used for all cases. In the analytical section, the difference between hospital bills and tariffs was first compared with different items, and hospital grouped specialties were then compared. It should be noted that in all analyses, the data was measured in terms of linearity. To carry out this study, an introduction letter was obtained from the Economics and Health Management Research Center to achieve the required information from the hospital and to perform the necessary legal steps for the study. In addition, necessary coordination was made with the management of Hazrate Rasoole Akram Hospital regarding access to cost information and hospital information system. In order to guarantee confidentiality, any tendencies and opinions were avoided at different stages of the research. Moreover, researchers committed to ensuring the confidentiality of the patients’ files and information; in this case, patients’ names were not recorded, and their file numbers were only extracted.

## Results

Overall, 59 different types of surgeries were performed in the studied hospital in the third quarter of the year, i.e. out of a total of 91 global surgeries in this period, 59 were carried out in Hazrate Rasoole Akram Hospital. The hospital had 1178 cases related to the global patients in the fall of 2017; 715 cases were related to global inpatients and 463 to global outpatients. Among the inpatients and outpatients who underwent global surgery, Social Security Organization (534 cases, 45.3%) and Public Health Insurance (338 cases, 28.7%) had the highest insurance cover respectively (Table 1).

**Table 1:** Frequency distribution of health insurance grouping of global outpatients and inpatients in the third quarter of 2017

Insurance type	Frequency	Percentage
Social security	534	45.3
Rural treatment services	129	11.0
Treatment services for other groups	12	1.0
Employee treatment services	68	5.8
Relief committee	56	4.8
Armed forces	28	2.4
Public health insurance	338	28.7
Foreigners–other groups	13	1.1

The most common surgeries in the third quarter were ophthalmic surgery (661 cases, 58%), general surgery (185 cases, 16%) and ENT surgery (129 cases, 11%). The least common global surgeries performed in this period included cardiac surgery (5 cases, 0.4%) and urology (8 cases, 0.6%). Moreover, the lowest number of bed day for global surgeries was 0.5 days for ophthalmic surgeries, while the highest one was related to neurosurgery (5 days) (Table 2).

**Table 2:** Frequency distribution of grouping- based surgeries performed in the studied period

Ward	No. of global surgeries	Frequency	%	Average hospital stay
Ophthalmic	14	661	7.57	0/5
General surgery	14	185	16	3
ENT	10	129	2.11	4
Obstetrics and Gynecology	11	126	11	3/8
Orthopedic	6	19	6.1	3
Neurology	1	12	1	5
Urology	2	8	0.6	3
Cardiac	1	5	0.4	4

Table 3 shows the overall hospital status in terms of outpatients and inpatients in the study quarter (39.3% outpatient surgeries and 60.7% inpatient surgeries). The average global bill for each outpatient surgery, the average actual bill for each case and total difference between global bills and actual bills was 395.2, 610.1 and 99528.8 Dollar respectively. For the hospitalized patients, the average global bill for each surgery, the average actual bill for each case and total difference between global bills and actual bills was 677, 1184 and 362,697 Dollar respectively. The total global bill for outpatients and inpatients was 642,423 Dollar; the total actual paid bill was 38,860,940,637; and total difference between global bills and actual p3073 Dollar. In addition, the franchise paid by the patients covered 8.7% of treatment costs, and subsidies paid by the Health Reform Department of the Ministry of Health, Treatment and Medical Education covered 8.9% of costs. Moreover, the insurer’s share of hospital costs for these patients was 41.4%. Unfortunately, patients had to pay the remaining 41% because no resource was defined for it.

**Table 3:** Summary of the amounts of actual bills and global bills in the studied quarter (Rials)

Admission type	Calculation type	No. of surgeries	Global bill				Actual bill	Difference between global bills and actual bills
			Total global bill	Franchise paid by patients	Insurer’s share	Ministry of Health subsidy	Total actual cost of hospitals with tariffs announced by the Ministry of Health	
Outpatient	Total surgeries	463	6,304,842,637	849,556,557	4,747,649,355	707,636,725	9,734,580,361	3429,737,723
	Average (per surgery)		13,617,371	1,834,895	10,254,102	1,528,373	21,025,012	7,407,641
Inpatient	Total surgeries	715	16,679,165,805	2,231,603,871	11,365,899,798	3,081,662,136	29,168,627,376	12,489,461,571
	Average (per surgery)		23,327,505	3,121,124	15,896,363	4,310,017	40,795,283	17,467,778
All surgeries (outpatient/inpatient)	Total surgeries	1178	22,137,730,823	3,042,900,807	16,098,505,226	3,802,664,885	38,860,940,637	15,913,703,073
	Average (per surgery)		19,511,043	2,615,586	13,678,734	3,216,722	33,024,794	13,513,752

Table 4 summarizes the general status of global surgeries at Hazrate Rasoole Akram Hospital in the third quarter of 2017. The highest frequency of global surgeries was observed in the ophthalmology department, accounting for 57% of all hospital surgeries. The highest and lowest global tariffs were respectively related to the gynecology and ophthalmology departments (**2050** and **118** Dollar). Calculations showed that the biggest and smallest difference between the global bills and the actual payment was related to the orthopedic (2418 Dollar) and ophthalmology (126) departments respectively. Finally, the difference between the global bills and the actual payment was calculated for all departments (results presented in the above table). The highest difference was related to the ophthalmology department (156,307 Dollar), while the lowest difference was related to the urology Sect. (2732 Dollar). General surgery, gynecology, ENT, orthopedic, neurology and cardiology departments were respectively the next. Finally, the total difference between the global bills and the actual payment at Hazrate Rasoole Akram Hospital was 461,806 Dollar for all departments.

**Table 4:** Patients’ record information in terms of surgery type at Hazrate Rasoole Akram Hospital in 2017 (Rials)

Ward	Descriptive statistics	Frequency of surgery	No. of hospitalized days	Franchise paid by patients	Insurer’s share	Ministry of Health subsidy	Total global bill
Total				3,042,900,807	16,098,505,226	3,802,664,885	22,947,237,564
Ophthalmology	Total	661		1,327,342,642	7,782,364,938	1,134,363,318	10,244,070,899
	Mean		1	2,005,235	11,732,370	1,715,883	15,453,489
	Maximum		11	15,616,715	22,605,365	18,139,737	49,917,932
	Minimum		0	180,203	0	0	4,056,800
General surgery	Total	185		884,827,274	4,125,916,479	1,476,388,247	6,487,132,000
	Mean		3	4,077,194	19,028,104	6,669,076	29,804,375
	Maximum		9	30,562,419	36,533,196	27,992,049	63,966,586
	Minimum		0	886,832	0	0	9,817,261
ENT	Total	129		166,495,142	1,355,975,049	302,002,078	1,824,472,269
	Mean		4	1,297,049	10,626,948	2,387,223	14,311,220
	Maximum		15	4,589,496	23,642,856	3,398,212	37,499,962
	Minimum		1	169,858	3,927,600	340,400	4,732,521
Gynecology	Total	126		476,449,016	2,071,238,870	624,679,157	3,175,533,688
	Mean		4	4,023,992	16,448,852	4,832,225	25,398,239
	Maximum		15	42,837,639	25,751,196	44,589,387	70,643,166
	Minimum		1	154,935	5,031,180	314,696	6,353,253
Orthopedic	Total	19		46,095,426	241,469,035	54,079,767	341,644,228
	Mean		3	2,421,497	13,474,181	2,629,993	18,525,671
	Maximum		12	7,803,460	31,743,346	14,242,123	51,395,886
	Minimum		0	606,640	3,844,584	164,402	6,980,392
Neurology	Total	12		83,131,255	293,365,583	105,768,287	482,265,125
	Mean		5	6,937,605	24,447,132	8,814,024	40,188,760
	Maximum		10	12,139,302	30,247,668	17,162,287	59,549,257
	Minimum		4	3,661,493	19,986,631	4,395,811	28,785,924
Urology	Total	8		14,319,966	94,589,064	16,769,208	125,678,238
	Mean		3	1,789,966	11,823,633	2,096,151	15,709,780
	Maximum		7	5,265,786	17,245,764	2,453,079	24,863,059
	Minimum		2	1,066,841	8,498,880	1,061,651	10,627,372
Cardiac	Total	5		44,240,086	133,586,208	88,614,823	266,441,117
	Mean		4	8,848,017	26,717,242	17,722,965	53,288,223
	Maximum		5	15,718,388	28,531,944	18,758,740	62,666,352
	Minimum		3	2,495,852	21,416,976	4,066,762	34,409,118

Operation room/technical fee	Lab/radiology	Bed day/nursing	Surgeon/surgeon’s assistant fee	Anesthesia	Other services	Total amount	Difference between total global and total services
1,461,044,700	516,020,084	6,216,957,136	3,150,183,415	4,705,375,001	22,137,730,823	38,860,940,637	15,913,703,073
710,529,008	36,992,256	3,174,466,134	463,113,020	2,245,806,080	9,390,884,939	16,531,721,437	6,287,650,538
1,071,676	44,490	4,786,229	685,992	3,391,949	14,174,822	24,924,706	9,471,218
2,081,072	7,556,024	11,566,800	14,411,760	7,806,400	44,162,169	59,013,337	28,652,003
380,800	0	952,000	0	0	0	5,338,800	-4,223,200
323,071,323	210,457,927	1,242,827,514	1,496,571,128	1,121,984,651	6,523,053,195	10,981,494,715	4,494,362,714
1,493,377	965,396	5,701,978	6,880,179	5,168,921	30,008,156	50,510,402	20,706,027
3,312,960	10,281,376	18,915,160	14,927,360	30,197,440	64,859,734	105,285,941	45,766,066
0	0	0	1,047,200	1,627,920	3,097,329	11,484,945	−1,156,495
124,057,024	23,844,744	543,359,712	350,867,500	518,935,200	1,672,329,889	3,276,495,269	1,452,023,000
968,993	195,571	4,281,702	2,734,698	4,052,022	12,977,675	25,547,330	11,236,110
2,006,816	2,221,016	10,618,608	17,081,900	10,472,000	47,341,962	74,576,887	41,478,731
9,520	0	1,142,400	0	857,800	4,198,220	8,240,269	1,778,092
248,621,642	156,182,556	968,897,961	621,513,335	603,906,431	3,305,893,206	5,943,577,631	2,768,043,942
1,977,430	1,210,148	7,583,083	4,922,167	4,795,105	26,516,011	47,305,117	21,906,878
5,616,800	8,955,128	23,499,500	10,091,200	8,834,560	81,595,565	110,588,833	50,853,810
390,320	218,960	1,552,900	952,000	0	2,148,423	12,900,987	388,886
17,074,120	42,421,088	99,385,600	60,528,160	76,921,600	402,925,925	708,692,493	367,048,265
979,778	2,353,281	4,837,023	3,471,338	4,176,016	20,366,271	36,605,520	18,079,849
2,370,480	13,547,912	18,634,800	9,710,400	8,529,920	93,992,017	134,731,425	83,335,539
0	0	0	913,920	1,570,800	6,038,956	10,231,700	3,015,008
21,412,384	37,795,352	110,365,676	80,402,112	80,444,000	460,242,258	460,242,258	313,567,457
1,784,365	3,149,613	9,197,140	6,700,176	6,703,667	38,353,522	38,353,522	26,130,621
1,804,992	6,947,696	16,469,000	11,361,168	10,757,600	57,038,103	57,038,103	38,055,193
1,766,912	654,024	4,659,760	5,243,616	3,284,400	30,791,740	30,791,740	18,511,650
7,339,920	3,440,528	40,379,640	32,520,320	25,818,240	107,916,685	107,916,685	94,137,095
917,490	430,066	5,047,455	4,065,040	3,227,280	13,489,586	13,489,586	11,767,137
952,000	1,374,688	10,870,300	5,026,560	4,332,080	20,347,745	20,347,745	19,869,279
847,280	120,904	3,105,800	2,703,680	1,865,9210	10,611,890	10,611,890	9,974,502
8,939,280	4,885,632	37,274,900	44,667,840	31,558,800	274,484,726	274,484,726	136,870,061
1,787,856	977,126	7,454,980	8,933,568	6,311,760	54,896,945	54,896,945	27,374,012
1,799,280	2,078,216	10,872,420	10,510,080	7,330,400	71,236,217	71,236,217	34,738,475
1,770,720	303,688	4,658,700	5,255,040	3,760,400	29,969,859	29,969,859	22,885,917

In this study, the dependent variable (Y) was the difference between the tariff and the bill which was precisely achieved from the difference between the actual bill for the patient and the global bill sent to the insurer. The information about the physician and insurance as well as bill items was considered independent variables. As shown in Table 5, in the outpatient ward at Hazrate Rasoole Akram Hospital, costs of the operating room, anesthesia and other services were significantly correlated with the difference between the actual and global bill; however, no significant relationship was found between the surgeon’s premium and the difference between the actual and global bill. Moreover, no significant relationship was found between the insurance companies and the difference between the actual and global bill. There was also no statistically significant relationship between the ophthalmic, orthopedic and ENT departments.

**Table 5:** The relationship between independent quantitative and qualitative variables with the difference between global tariffs and cost bills as dependent variables using linear regression in outpatient surgeries

Variable	Coefficient	T	P > t
Operating room costs	3.34986	9.54	0.000
Surgeon’s fee	.0636388	1.43	0.155
Anesthesia fee	.4641438	6.45	0.000
Costs of other services	.3108476	15.26	0.000
Basic insurance organization			
Public Health Insurance	_162436.1	−1.26	0.207
Relief Committee, Rural Health Services and f oreigners	113,724.6	0.85	0.397
*Armed Forces	165,744.2	0.56	0.578
Treatment Services for employees and other groups	_58748.57	−0.33	0.740
Grouping the operation type			
Ophthalmology	_302557	−0.70	0.483
ENT	_216804	−0.43	0.670
Orthopedic	_642940.2	−1.00	0.316

As shown in Table 6, there is a signif6icant relationship between independent variables such as operating room costs, nursing services, anesthesia fees and costs of other services and the difference between actual and global bills. Among the insurance companies, Relief Committee, rural health services and foreigners had a significant relationship with the difference between actual and global bills. Moreover, a significant relationship was found between surgeries performed in gynecology, ophthalmology, and ETN departments and the difference between actual and global bills.

**Table 6:** The relationship between dependent quantitative and qualitative variables with the difference between global tariffs and cost bills in inpatient surgeries

Variable	Coefficient	Std. error	T	P > t
Operation room costs	1.813807	.3112659	5.83	0.000
Bed day costs	.0931373	.1097859	0.85	0.397
Surgeon’s fee	−.0819103	.0578948	−1.41	0.158
Nursing services	12.95004	2.235872	5.79	0.000
Anesthesia fee	.5926864	.061837	9.58	0.000
Costs of other services	.3273084	.0114789	28.51	0.000
Basic insurance organization				
Public health insurance	−411,840.2	236,216.2	−1.74	0.082
Relief Committee, rural health services and foreigners	−526,569.1	309,867.6	−1.70	0.090
Armed Forces^a^	−399,919.5	722,003.5	−0.55	0.580
Treatment Services for employees and other groups	−186,564.5	468,382	−0.40	0.691
Grouping the operation type				
Gynecology	1,390,989	386,412.1	3.60	0.000
Ophthalmology	1,058,021	310,024.2	3.41	0.001
ENT	951,381.8	492,320.6	1.93	0.054
Urology, orthopedic, cardiac and neurology	46,669.77	484,491.7	.10	0.923

^a^In the section “basic insurance organization, social security insurance was used as the basis of regression, and in grouping the surgery type, general surgery was considered the basis of regression

## Discussion

This study aimed to compare the global surgery tariffs with the actual cost in Hazrate Rasoole Akram Hospital in the third quarter of 2017. 463 outpatient files and 715 inpatient files were studied. The highest rate of health insurance coverage for people undergoing global surgery in this period was related to the Social Security Organization (45%). The majority of global surgeries were related to ophthalmic surgeries (57%). In this surgical group, the hospital lost 276 Dollar per case. The findings of this study are inconsistent with the results of a study conducted by Hosseini et al. who showed that ophthalmology department, with an 18% share out of total global surgeries, was profitable in 99.6% of the cases [16]. General surgery (16%) accounts for the second largest number of global surgeries. The average actual bills in the general surgery ward were 601 Dollar higher than the global bills and the average hospital stay was 3 days. In a study carried out by Radin Manesh, the average stay in the general surgery ward was 4.1 and 5.6 days in Hospital A and B respectively. Compared to the present study, it is likely that different hospitals have different global surgeries, or some hospitals are better at reducing hospital stays due to better patient management. His results also revealed that hospital stays might differ significantly for identical surgeries. For example, the average number of stay for “total thyroidectomy” was 5 days In Hospital A, while it was 7.8 days in Hospital B. Concerning “cholecystectomy”, the number of hospital stay was 4 and 5.8 days in Hospital A and Hospital B respectively. It was the case for other surgeries. Therefore, it can be said that hospital policies and management are fundamentally different in various hospitals. The study also found that there was a large discrepancy between the actual costs and global tariffs in the general surgery wards of both hospitals, and that the hospitals lost a considerable amount of money in this regard [15]. The results of the study by Hosseini et al. are not in line with the results of the present study; they stated that, in the general surgery ward with a 20% share out of total surgical procedures, the cost difference was profitable in 92% of the cases (Rahil Hosseini). The results of this study depicted that, in some cases, the difference between the hospital stay and the standards determined in the global system was 3 to 5 days and less than the standard level [16].

Concerning the difference between the average hospital cost and the global tariff, Chatruz et al. found out that the cost of surgical procedures was 3 to 312% higher than the approved global tariff in all cases (61 cases) except for 7 surgeries, with the highest difference (312%) being related to septoplasty. This difference was more than 50% [9] in 22 surgical procedures; the final result of this study was similar to the results of the study conducted at Rasoole Akram Hospital.

Contrary to the findings of the present study, Hosseini et al. concluded that the cost of global surgery was beneficial for the hospital in 86% of the cases (96% of the differences were significant). They also found that the average hospital stay was less than the standard hospital stay in more than 99% of the cases (it was significant in 64% of the cases) [16].

Owing to the decreased length of stay in global surgical procedures, revision of the reimbursement system seems necessary, and a prospective reimbursement system must be implemented for other diagnoses and surgical procedures.

The results showed that there was a significant relationship between independent variables such as costs of the operating room, nursing services, anesthesia and other services and the difference between the actual bill and the global bill; these results were consistent with the results of a study carried out in two hospitals affiliated to Tehran University of Medical Sciences. In his study in 2013, Radin Manesh found that there was a significant and positive relationship between costs of medicine and consumables, the operating room and surgery and the difference between tariffs and bills. He also showed a significant and positive relationship between the hoteling and paraclinical costs and the difference between tariffs and bills [15]. In other words, as the above-mentioned independent variables increased, the difference between the actual bill and the global bill decreased, and vice versa. It should be noted that the significance of variables indicates a strong relationship between these types of relationships.

Among the insurance companies, the Relief Committee, Rural Treatment Services and Foreigners had a significant relationship with the difference between the actual bill and the global bill of inpatients. Studies on insurance status and its relationship with the differences between the global and actual bill in two different hospitals showed that patients with Iranian Insurance cover experienced a greater difference in tariffs and bills [15]. Surgical procedures performed in gynecology, ophthalmology and ENT departments had a significant relationship with the difference between actual and global bills. Comparing inpatient and outpatient departments revealed that the type of insurance had a different role and relationship with the difference between the actual and global bill in both departments, and that the most important reasons for this difference included increased length of stay, increased hospital deductions, increased patient’s costs for the hospital and thus decreased payments by the insurance companies.

The prevailing reimbursement system in Iranian hospitals is often based on the retrospective reimbursement method, and only a limited number of medical procedures use a prospective (global) payment system. According to the results of the present study, the hospital global bill was less than the patient’s actual bill, indicating that some components must be taken into account in determining the expenses and tariffs for Tehran referral university hospitals that usually treat complex patients.

In his study, Margani showed that there was a significant difference between the costs of medicine and medical consumables and anesthesia and the bill cost of each surgery in the hospitals owned by Tehran Social Security Organization [17]. Godari et al. revealed in their study that the costs of surgical procedures for hospitals increased over the years 2004–2014 [18]. In their study, Hosseini et al. concluded that global surgery was beneficial in 86% of the cases [16]. A study conducted at Tehran University of Medical Sciences by Chatruz et al. showed that 61 cases (out of 68) of global surgical procedures were detrimental, and hospitals experienced no loss only in 7 cases [12]. Reviewing previous studies, it is clear that large specialized and sub-specialized hospitals are more susceptible to losses resulted from the difference between global tariffs and actual bills due to the admission of patients with more complex medical problems, increased length of stay, higher hoteling costs and higher costs of consumables; therefore, they need to revise their global tariffs proportional to the increasing costs.

Comparing the cost of global surgery with the actual cost at the Cancer Institute, Arab et al. stated that there was a significant difference between the cost of global surgery and its actual cost in each of the years 2003 and 2004 (actual costs were far more than global costs) [19]. In his study in Hormozgan, Hosseini acknowledged that the cost difference in 1286 cases was beneficial to the hospital in 86% of the cases [16]. In a similar study conducted in one of the hospitals in Bushehr in 2001, Omrani Khoo stated that the bill cost of 570 (out of a total of 1667 patients) patients (34.2%) was higher than their approved tariffs, and the approved tariff of 1097 patients (65.8%) was higher than their bill costs; the difference between bill costs and global tariffs was significant [20].

Sharifian also regarded this point as one of the disadvantages of the global payment system. He mentioned that some variables such as age, sex, presence or absence of complications and associated diseases, specific level of complications and associated diseases, infant’s birth weight and infant’s weight at the time of admission did not exist in this system. In addition, the severity of the disease/patient’s clinical complexity level and the risk level of death cannot be determined with respect to this system [21]. In recent years Iran made greater investments in health care and its core outcomes than most comparable nations [22] and To better manage these finance resources the structure of Iran’s health system, types of basic and complementary insurance policies, the payment system, the people’s consumption and payment pattern, the severity of diseases and patient’s clinical complexity level should be taken into account in determining health service tariffs [10]. Tariffing is a process which must be taken into consideration through negotiations between health system trustees, insurance agency representatives, and service provider representatives. The reimbursement method of medical expenses is effective in the financial management and control of hospital costs. One of the essentials of the health system in Iran is to transform the global system into a case system based on indigenous diagnostic groups [23].

## Conclusion

The prevailing reimbursement system in Iranian hospitals is often based on a retrospective method, and the prospective (Global) payment system is only used in a limited number of medical procedures. According to the results of the present study, the amount of the global bill in the study hospital was less than the actual bills of the patients, indicating that special attention must be paid at the time of setting tariffs for the referral university hospitals in Tehran that usually treat complex patients.

It seems that the studied hospital should make a substantial revision of the costs of this type of surgeries and, if possible, intervene in this regard. In addition, a large amount of the bill costs has been recorded in other items and it has caused these costs to be unclear. Therefore, it is recommended that medication, appliances and other items be registered in their departments so that they can be traced easily and be transparent. Moreover, based on this study and previous studies, it has been found that in Tehran university hospitals which generally treat complex patients with underlying diseases, the global tariff does not meet the costs and should be taken into account when setting the tariff. It should be borne in mind that when there is such a high discrepancy between the global bills and the actual hospital bills, it will in the long run bring a great deal of financial burden and inevitably need to be credited with other sources; as university hospitals normally face budget deficits and try to take this amount from their patients, it could raise the issue of high out-of-pocket payment which in turn brings up other issues.

## Data Availability

The datasets used and/or analysed during the current study are available from the corresponding author on reasonable request.
